# Effects of Stochastic Inputs on Calcium-Dependent Synaptic Plasticity

**DOI:** 10.1186/1471-2202-12-S1-P8

**Published:** 2011-07-18

**Authors:** Harshit S Talasila, David A Stanley, Berj L Bardakjian

**Affiliations:** 1 Electrical and Computer Engineering, University of Toronto, Toronto, Ontario, M5S 3G4, Canada; 2 Department of Bioengineering, Arizona State University, Tempe, Arizona, 85281, USA; 3 Institute of Biomaterials and Biomedical Engineering, University of Toronto, Toronto, Ontario, M5S 3G9, Canada

## 

Activity dependent potentiation and depression in synaptic efficacy is thought to be one of the underlying mechanisms of learning and memory. Recent studies have shown that moderate increase in synaptic spine calcium concentration leads to synaptic depression, while large increase results in potentiation [1]. We produced a computational model of a small CA3 hippocampal network of cells governed by calcium dependent plasticity developed by [2] to investigate the effects of i) stochastic network inputs (SNI) and ii) spontaneous cell activation (SCA) on the Ca dependent model of plasticity.

We used a small hippocampal network model consisting of 72 pyramidal cells and 16 interneurons, where each cell was a modified version of the Traub model [3,4]. The calcium-dependent plasticity model was implanted to govern the changes in synaptic efficacy between the cells. The presence of spontaneous activation of AMPA, NMDA, GABAA and GABAB synaptic elements were varied between zero (low) and 5,5,3,3 Hz (high) respectively. The cells were also provided with SNI that had a Poisson distribution with an expected value of spike per second of either 10(low) or 100(high). The effects of the rate of, SNI and SCA on synaptic plasticity were studied.

**Figure 1:**
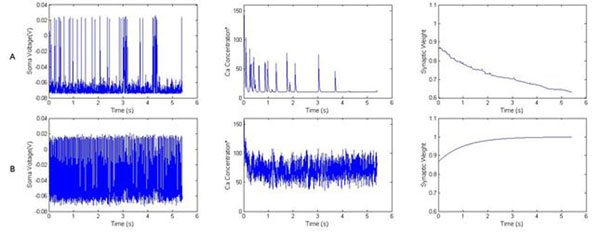
Effects of SNI and SCA on membrane voltage (left), intracellular calcium concentration (middle) and synaptic weight of AMPA synapse (right), for case **A)** high SCA and low SNI, **B)** low SCA and high SNI. (*Calcium Concentration has arbitrary units, following the convention used in [3]).

We observed that the rate and direction of synaptic weight change is strongly affected by the rate of SNI, with a high input rate increasing the internal calcium concentration resulting in an increase in synaptic weight (Figure 1B), while a lower rate only raised the calcium concentration moderately (Figure 1A), resulting in decrease in synaptic weight. Furthermore, the rate of synaptic weight change is affected by SCA. Under low SNI conditions, low SCA provides a higher rate of change, while under high SNI conditions it actually reduces the rate of change. These results demonstrate that the rate of stochastic inputs to a cell plays a role in determining its direction of plasticity (potentiation or depression), while the cell’s spontaneous activations influences its rate of change.
